# Myeloid tumors accompanying systemic mastocytosis, basophilia, and abnormal platelet-derived growth factor receptor β: A case report

**DOI:** 10.1097/MD.0000000000024707

**Published:** 2021-02-26

**Authors:** Yanfen Li, Yu Jing, Hua Wan, Daihong Liu

**Affiliations:** a Department of Hematology, Chinese PLA General Hospital; b Beijing USCI Medical Laboratory, Beijing, P.R. China.

**Keywords:** basophilia, fusion gene, platelet-derived growth factor receptor β, systemic mastocytosis, thrombocytosis

## Abstract

**Introduction:**

Myeloid neoplasms with platelet-derived growth factor receptor β (PDGFRB) rearrangement usually present with eosinophilia in the peripheral blood and bone marrow. Here we report a case of systemic mastocytosis related myeloid neoplasms with basophilia and PRKG2–PDGFRB fusion gene.

**Patient's concerns:**

A 53-year-old male patient felt fatigue with thrombocythemia and normal hemoglobin over 2 years. Considering the possibility of primary thrombocytosis, the patient was treated with hydroxyurea and interferon. Then the therapy was stopped due to adverse events and worsen condition.

**Diagnosis:**

Acute myelogenous leukemia (AML) diagnosis was confirmed by bone marrow morphology and flow cytometry. PDGFRB rearrangement was detected by fluorescence in situ hybridization (FISH) test, with chromosome karyotype 46,XY,t(4:5)(q21:q33). PRKG2–PDGFRB fusion was observed by next generation sequencing (NGS) and verified by RT-PCR followed by Sanger sequencing. The results of bone marrow aspiration, bone marrow biopsy, and immunophenotyping showed systemic mastocytosis-related myeloid tumor with basophilia.

**Interventions:**

Imatinib 400 mg/d was given on the day of admission. Azacitidine 75 mg/m^2^ was given for induction therapy for 10 days, and followed by one course of DHAG consolidating therapy. Imatinib was taken orally continuously.

**Outcomes:**

On the 8th day of treatment, the patient's diet and fatigue improved. The hematological and bone marrow morphological remission was achieved on the 25th day. Cytogenetic complete remission was achieved 3 months later and continued to present (December 20, 2020). PRKG2–PDGFRB fusion gene turned negative 7 months later from diagnosis.

**Lessons:**

Patients with increased basophilic granulocyte and/or mast cells in peripheral blood and/or bone marrow should be screened for PDGFRB abnormality and myeloid or lymphatic tumor. Patients bearing PDGFRB abnormality have a good response to imatinib.

## 1 Introduction

Myeloid and lymphatic tumors, which are accompanied by eosinophilia and rearrangement of platelet-derived growth factor receptor α (PDGFRA), platelet-derived growth factor receptor β (PDGFRB), and fibroblast growth factor receptor 1 (FGFR1), are classified as an independent disease type in 2008 World Health Organization classification of hemolymph tumors. PDGFRB is a kind of tyrosine kinase receptor which is located at 5q32 of chromosome 5. When activated, it could regulate the downstream signal pathways including PI3K-AKL-MTOR and RAS-RAF-MAPK and participate in life activities such as embryonic development, angiogenesis, and proliferation, differentiation, and migration of cells.

Abnormality of PDGFRB gene is associated with chronic myeloid leukemia (CML), acute myelogenous leukemia (AML), chronic myelomonocytic leukemia (CMML), acute lymphocytic leukemia (ALL), chronic eosinophilic leukemia (CEL), myeloproliferative disease (MPD), and other diseases. At least 30 genes are reported to fusion with PDGFRB gene.^[1]^ Following further learning of this kind of tumor and the application of next sequencing technology, more fusion partner genes of PDGRFB gene are found, and different fusion gene types may have different clinical manifestations.

## 2 Case report

The patient is a 53-year-old man. Prior written and informed consent was obtained from the patient and the study was approved by the Ethics Committee of Chinese People's Liberation Army General Hospital. In July 2017, the blood platelet count was 395 × 10^9^/L when he underwent left parotid gland Warthin operation. The white blood cells (WBC) was 7.35 × 10^9^/L and hemoglobin was 139 g/L at that time. While the ratios of eosinophils and basophil were 0.037 and 0.008, respectively. On April 27, 2018, the patient showed fatigue and discomfort of precordial area. The blood routine examination showed WBC 7.5 × 10^9^/L, hemoglobin 141 g/L, and blood platelet count 376 × 10^9^/L. The proportion of leukocyte in each system was normal and there was no abnormality in echocardiography and electrocardiograph.

On October 15, 2018, the patient's blood routine examination showed blood platelet count 552 × 10^9^/L when diagnosis of appendicitis. Bone marrow biopsy showed erythroid and granulocytic proliferation in hematopoietic tissue and the ratio between erythroid cells and granulocytic cells was normal. A few of granulocyte precursor cells were observed. And neutrophilic metamyelocytes, metamyelocytes, and cells in following stages were observed dispersedly or in piles. Whereas erythroid cells were mainly observed in intermediate and late stages, separately distributed or in piles. These cells in primordial and early stage were few observed. Four to 6 megakaryocytes, most of which were hypersegmented neutrocytes, could be found in per vision of high-power lens. Lymphocytes and plasma cells were found without fibrosis. All these were the characteristics of myeloproliferative changes, not the typical characteristics of primary thrombocytosis. JAK2/V617F, CSF3R, MPL, and CALR mutations were detected negative. The cytogenetic analysis results were: 46, XY, t(4:5)(q21;q33)[7]/46, XY[13]. No treatment was followed.

Then, fatigue continued to worsen. Since December 2018, antinuclear antibodies (1:320 positive centromere) and anti-centromere antibodies tests were positive in several testing. So autoimmune related disease was considered and patient was treated with hydroxychloroquine for 3 months. However, the fatigue did not remit, and the therapy was stopped. At the end of March 2019, the blood routine examination showed that blood platelet count 1660 × 10^9^/L, WBC 13 × 10^9^/L, hemoglobin 137 g/L, and basophilic granulocytes 0.65 × 10^9^/L. The patient refused to take bone marrow aspiration. Considering the possibility of primary thrombocytosis, the patient was treated with hydroxyurea and interferon. As a result, the blood platelet count declined to 890 × 10^9^/L. However, interferon was discontinued due to cutaneous pruritus and rash. The patient gradually appeared dull complexion, aggravation of fatigue, fever, and difficultly in walking.

**Figure 1:**
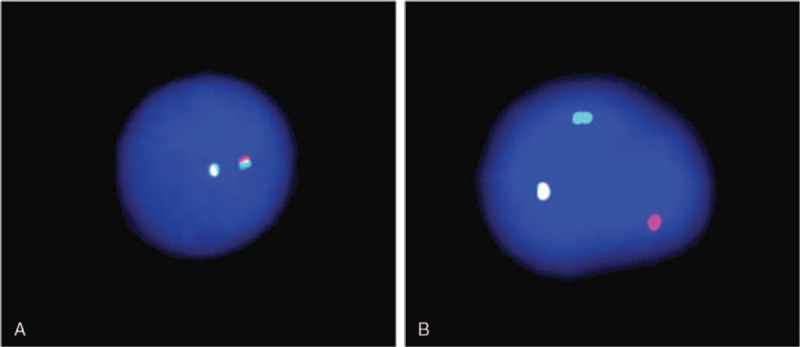
Fluorescence in situ hybridization (FISH) test. Red and green signals were labeled on the 5′-end and 3′-end of PDGFRB gene, respectively. (A) The normal cells showed yellow signals or red and green signals in close tandem, (B) while the pathological cells showed separated red and green signals. PDGFRB = platelet-derived growth factor receptor β.

The patient was admitted to our hospital 3 days later after bone marrow fluorescence in situ hybridization (FISH) test on December 16, 2019. PDGFRB gene rearrangement with a positive rate of 87% was discovered in the test (Fig. 1). Routine blood examination showed: WBC 25.48 × 10^9^/L, hemoglobin 66 g/L, blood platelet count 303 × 10^9^/L, ratio of lymphoblast and prolymphocyte 45%, and peripheral blood basophils 2 × 10^9^/L. Abdominal ultrasound showed an enlarged spleen, about 17.1 cm long and 5.6 cm thick. The ECOG score was 4. On that very day, the patient was treated with imatinib 0.4 g/d. Acute myeloid leukemia was diagnosed by bone marrow morphology and immune typing later. Azacitidine (75 mg/m^2^) was added to the therapy.

**Figure 2:**
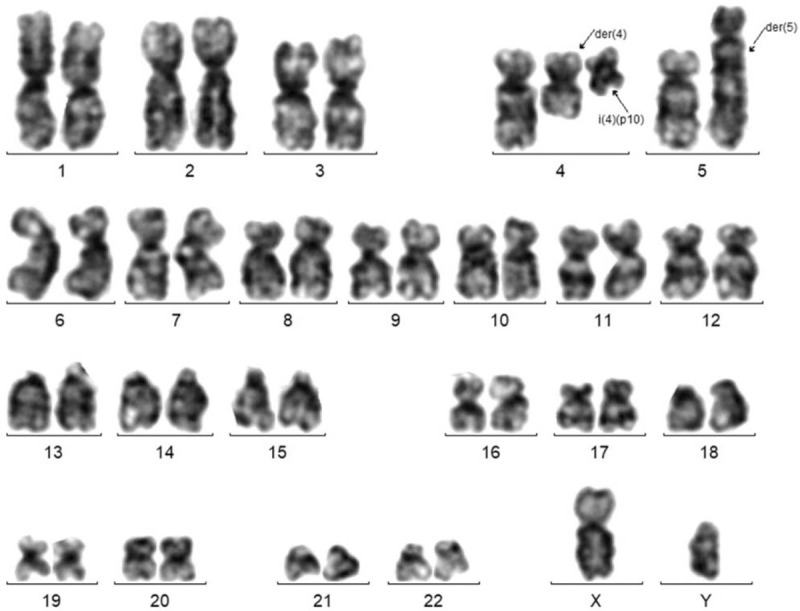
Representative G-banded karyotype showing 47,XY,+4,i(4)(p10),t(4;5)(q21;q33).

**Figure 3:**
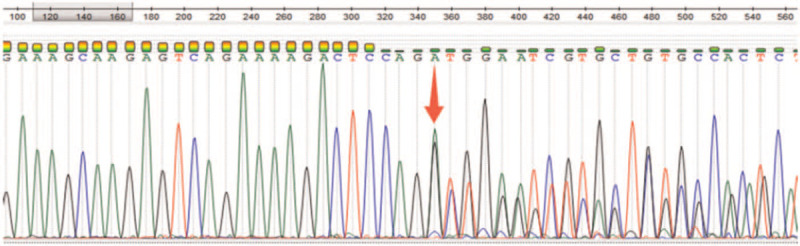
Sanger sequence of PRKG2–PDGFRB fusion. The arrow shows the breakpoint and left part is partial of PRKG2 gene. PDGFRB = platelet-derived growth factor receptor β.

The results of bone marrow aspiration showed that bone marrow hyperplasia was extremely active, account for about 20% of the original cells. In addition, about 45.2% of the cells were medium-sized, round, or fusiform. The cytoplasm was filled with thick black purple granules, most of which looked like mast cells, and a few of which looked like basophils. There were 1322 megakaryocytes in the whole smear, with various types visible. Blood platelets were visible in piles. Blood tablet was account for 34.0% of the original cells, and basophilic cells were increased. Based on these results, the patient was considered as acute myelogenous leukemia with increased basophilic cells and mast cells. Whereas bone marrow biopsy showed hyperactivity of bone marrow hyperplasia with an increased proportion of mast cells scattered or clustered (>15 mast cells/aggregation sites). Immunohistochemical test showed: CD34+ partial, CD117+, CD2–, CD25+, CD56+ individually. Thus, systemic mast cell hyperplasia with associated hematopoietic tumor was considered. Immunophenotyping showed that the proportion of myeloid primitive cells and mast cells increased, which accounted for 18.53% of nuclear cells. These cells expressed CD25 and showed abnormal phenotype. Beside, basophils were observed saliently. The karyotype was: 46,XY,t(4;5)(q21;q33)[5]/47,XY,+4,i(4)(p10)(4;5)(q21;q33)[15] (Fig. 2). PRKG2–PDGFRB fusion was found positive with an abundance of 95.7% according to the next generation sequencing (NGS) data, and confirmed by RT-PCR followed by Sanger sequencing (Fig. 3). In addition, RUNX1 mutation was also observed by NGS. Therefore, the patient was diagnosed as systemic mastocytosis-related myeloid tumor with basophilia and PDGFRB abnormalities.

**Figure 4:**
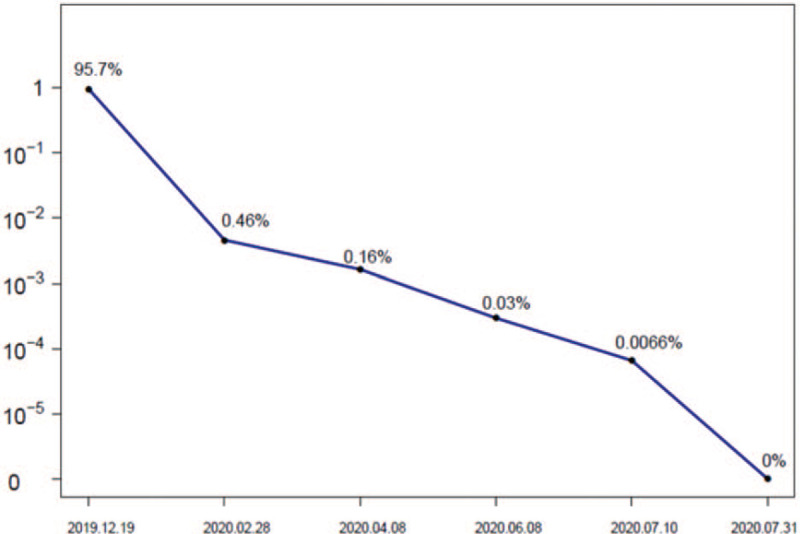
Minimal residual disease (MRD) assessment.

On the 8th day of treatment, the patient's diet and fatigue improved. Azacitidine was continuously used for 10 days. The hematological and bone marrow morphological remission was achieved on the 25th day. Then a course of DHAG consolidating therapy was conducted, and imatinib was taken orally continuously. Cytogenetic complete remission was achieved 3 months later and continued to present (December 20, 2020). The disease free survival was 12 months. PRKG2–PDGFRB fusion gene turned negative 7 months later from diagnosis (Fig. 4). Antinuclear antibody (1:320 positive for centromeric type) and anti-centromeric antibody tests were positive and no autoimmune disease-related treatment was given during this period.

## 3 Discussion

Myeloid tumors with PDGFRB rearrangement are usually characterized by eosinophilia in the peripheral blood and bone marrow. Patient presented mild thrombocythemia in the first 1 year, which was the chronic phase of the disease. The onset is insidious. After a significant increase in platelets, the basophil ratio in peripheral blood elevated. However, basophilia may also occur on patients with myeloproliferative diseases, which leads to misdiagnosis. Therefore, 2 years had passed for this patient from the clinical symptoms to the confirmed diagnosis. During this period, myeloid leukemia progressed from atypical thrombocythemia to myelodysplastic syndrome/myeloproliferative disease and then to acute myelogenous leukemia. PRKG2–PDGFRB fusion gene formed by t(4;5) translocation may played an crucial role. This is the first case that the fusion was confirmed simultaneously by NGS, FISH, cytogenetic analysis, and Sanger sequence. In addition, in total of 6 cases have been reported with PRKG2–PDGFRB fusion or t(4;5) translocation.^[2–7]^ Among all the 7 cases, 5 patients were reported to be with peripheral blood basophil proliferation, 4 patients with systemic mast cell hyperplasia, and 6 with mast cell hyperplasia (Table 1). We inferred that patients with PRKG2–PDGFRB fusion gene are more likely to present peripheral blood basophil proliferation or systemic mast cell hyperplasia. More cases are required to verify this speculation.

**Table 1:** Summaries of reported cases with hematologic neoplasm associated with t(4;5)(q21;q33), *PRKG2/PDGFRB* fusion gene.

Studies	Age/Sex	Laboratory finding	PE/Image finding	Initial diagnosis	Genetic finding	Treatment	Outcome
Wang RC et al^[2]^	59/M	Anemia, thrombocytopenia and high tryptase level (250 ng/mL); basophilia, 40%	Small nodule in bilateral lung; small LAP in mediastinum, retroperitoneu m, and inguinal region;Splenomegaly hepatomegaly	Mast cell leukemia with peripheral basophilia	t(4;5)(q21.3;q33); PDGFRB rearrangement detected by FISH	C/T with idarubicin and cytosine arabinoside, then shifted to Imatinib 400 mg twice daily	In complete remission 33 months after diagnosis
Brown LE et al^[3]^	26/F	Basophilia, 37%, 4.1 k/μL; serum tryptase level of 175.0 ng/mL	Cachexia; massive splenomegaly	SM-AHN	t(4;5)(q21;q33); abnormalities of PDGFRB	Imatinib, 200 mg/d	Complete hematologic remission 6 months after initiation of imatinib therapy
Walz C et al^[4]^	45/F	Leukocytosis of 34 × 10^9^/L with basophilia of 20–40% serum tryptase: 138 ng/mL	Splenomegaly	Chronic myeloproliferative disorders	t(4;5;5)(q23;q31;q33)[20]	Imatinib, 400 mg/d	Complete hematologic responses within 3 and 4 weeks, complete cytogenetic response was seen after 7 months of treatment
Lahortiga I et al^[5]^	61/M	WBC 26,300/mm^3^, with 26% basophils; serum tryptase 200 ng/mL	Splenomegaly	Indolent systemic mastocytosis with chronic basophilic leukemia	t(4;5)(q21.1;q31.3); PDGFRB rearrangement detected by FISH	Imatinib 200 mg/d, then increased to 300 mg/day	Cytogenetic remission in 1 year after diagnosis; in Cytogenetic remission for 3 years after imatinib initiated.
Gallagher G et al^[6]^	45/M	Anemia and eosinophilia, 1.1 × 10^9^/L	Extensive intraperitoneal and retroperitonal LAP; splenomeglay	Atypical myeloproliferative disease	t(4;5)(q21;q33); PDGFRB rearrangement detected by FISH	Imatinib, 100 mg/d after failure to response with INFa, cladribine	Complete morphological and molecular remission in 10 months after induction of imatinib.
Bakul I. Dalal et al^[7]^	45/M	Hb 10.8 g/dL; WBC 6500/mL, absolute eosinophil count 1100/mL, normal liver function tests, normal LDH (59 U/L)	Hepatomegaly splenomegaly retroperitoneal LAP	Aggressive systemic mastocytosis	t(4;5)(q21.3; q33)[20]	Imatinib mesylate (100 mg/d) after failure of IFN a2b and prednisone, and four monthly cycles of 2’chloro-deoxyadenosine	Complete disappearance of symptoms with resolution of the hepatosplenomega ly and LAP in weeks; Cytogenetic testing on this same marrow sample showed only 1 of the 25 metaphases to exhibit abnormality
Present case	53/M	fatigue, thrombocytosis	Splenomegaly	SM-AHN	47,XY,+4,i(4)(p10),t(4;5)(q21;q33); PDGFRB rearrangement detected by FISH; PRKG2-PDGFRB fusion detected by NGS	Imatinib 400 mg/d +Azacytidine 75 mg/m^2^, DHAG consolidation therapy	Complete morphologically in 3 months and molecular remission by PCR in 7 months after induction of imatinib.

FISH = fluorescence in situ hybridization; SM-AHN = systemic mastocytosis associated with hematological neoplasm; PDGFRB = platelet-derived growth factor receptor β.

Although reactive mast cell hyperplasia existed in myeloid and lymphatic tumors such as acute myeloid leukemia, lymphocytic lymphoma, and chronic lymphocytic lymphoma, it should be distinguished from systemic mast cell hyperplasia. For systemic mast cell hyperplasia, it is mainly diagnosed by bone marrow biopsy for pathology, which is characterized by multifocal, compactly, and clustered rather than dispersedly clonal proliferation of cells around the blood vessels and trabecular bone. In addition, systemic mast cells also express CD25 and/or CD2, beyond mast cell markers CD117 and trypsin. The rates of KITD816V mutation are as high as 80% in patients with systemic mastocytosis. However, it is not indispensable for diagnosis.^[8,9]^ In this report, the patient was observed with multiple, dense mast cell infiltrates in the bone marrow, and >25% spindle or fusiform mast cells were observed on bone marrow smear. In addition, mast cells in bone marrow also expressed CD25 in this case. In conclusion, this patient met 1 major diagnostic criterion and 2 minor diagnostic criteria for systemic mast cell hyperplasia. Besides myeloid and lymphatic tumors bearing eosinophilia and PDGFRB rearrangement, PDGFRB rearrangement also could occur in a portion of Ph-like ALL patients.^[10]^ NGS technology could identify the types of fusion gene and provides a reliable basis for disease diagnosis and accurate treatment.

The prognostic significance of RUNX1 mutation in this patient is unclear. Pardanani et al^[11]^ summarized 580 cases with systemic mast cell hyperplasia and suggested that mutations of ASXL1, RUNX1, and NRAS were associated with poor prognosis. However, mutations of RUNX1 always occur in SM-AHN at a mutation rate of 6%. And its meaning depends on the effect of RUNX1 in related clonal hematological diseases. Baer et al^[12]^ summarized the results of 61 patients with myeloid and lymphatic tumors accompanying PDGFRA, PDGFRB, FGFR1, or PCM1-JAK2 rearrangements. RUNX1 mutations occurred in 5 patients, including 1 patient with PDGFRA rearrangement and 4 patients with FGFR1 rearrangement, which suggested that RUNX1 mutations were associated with poor prognosis (myeloid/lymphoid neoplasms associated with eosinophilia and rearrangement of PDGFRA, PDGFRB, FGFR1, or PCM1-JAK2). However, RUNX1 mutation has not been reported co-occurring with PRKG2–PDGFRB fusion,^[2–7]^ leaving its significance to be further studied.

The treatments of hematopoietic tumor associated with systemic mast cell hyperplasia focus mainly on related hematopoietic system tumors. For myeloid tumor patients with PDGFRB fusion gene, imatinib was associated with long lasting and long-term remission. Chan et al^[9]^ reported that 26 patients with myeloid tumor bearing PDGFRB fusion achieve durable long term remissions with imatinib, with a 10-year overall survival of 90% and a 6-year PFS of 88%. The patient in this report was weak, and was not suitable for routine chemotherapy. Chemo-free imatinib + azacytidine were chose and followed by 1 course of consolidating therapy based on AML protocol. This treatment achieved great success. However, it was unclear when to stop the drug. Sequential allogeneic hematopoietic stem cell transplants may follow, once minimal residual disease (MRD) levels increased.

Moreover, the patient was positive at a low titer of anti-centromere antibody test and there was no significant change after the systematic treatment. It may due to that anti-centromere antibody is not unique to autoimmune diseases. It could occur in some normal people.^[13]^ Till now, there are no reports of AML patients with positive of anti-centromere antibody. Therefore, no continued treatment for autoimmune disease was arranged for the patient. A close follow-up continued.

## 4 Conclusion

Imatinib is an effective therapy for patients with PDGFRB abnormality. Patients with increased basophilic granulocyte and/or mast cells in peripheral blood or bone marrow should be screened for PDGFRA/PDGFRB abnormality and myeloid or lymphatic tumor. NGS technology could identify the specific and unknown fusion genes and provides a reliable basis for disease diagnosis and accurate treatment.

## Acknowledgments

The authors thank the patient for consenting to participate. They thank all the reviewers and editors for their suggestions on this manuscript.

## Author contributions

**Supervision:** Yu Jing, Daihong Liu.

**Writing – original draft:** Yanfen Li.

**Writing – review & editing:** Yu Jing, Hua Wan, Daihong Liu.
